# Supplementary material for: Functional Dissociation of θ Oscillations in the Frontal and Visual Cortices and Their Long-Range Network during Sustained Attention
Source: eNeuro. 2019 Nov 13;6(6):ENEURO.0248-19.2019. doi: 10.1523/ENEURO.0248-19.2019 (PMC6860984; doi:10.1523/ENEURO.0248-19.2019)
Supplement: Extended Data Figure 3-1 — GLM analysis of CFC. To dissect the confounding effect of θ and attention on MI, MIs were further analyzed through GLM with three predictors: θ amplitude, attentive state, and two-way interaction of θ amplitude and attentive state. As a result, we found statistically significant main effect of θ amplitude on θ-low-γ coupling in both regions (frontal: t = 14.529, p < 0.001, visual: t = 9.902, p < 0.001) and on θ-high-γ coupling in the visual (t = 2.648, p < 0.01). The effect of θ amplitude on frontal θ-high-γ coupling did not reach statistical significance (t = 1.747, p = 0.081). Also, the effect of attentional state on MI was statistically significant only for visual θ-low-γ coupling (t = –2.864, p < 0.01) with non-significant effect on the other couplings (|t|s < 1.688, ps > .091). Most importantly, the two-way interaction between θ amplitude and attentional state was statistically significant for θ-low-γ coupling in both regions (ts > 2.294, ps < 0.05), but not for θ-high-γ coupling (|t|s < 1.301, ps > .193). Such two-way interaction with positive slope value (ß = 1.0 × 10^–6^ for frontal, ß = 1.3 × 10^–6^ for visual) suggests the effect of θ amplitude on θ-γ coupling becomes stronger with increased level of attention, especially for θ-low-γ coupling in both regions. In short, the change of CFC observed in this study can be best explained by a synergetic effect of θ amplitude and attentional state, rather than by single factor. Download Figure 3-1, PDF file. [file sup_enu-eN-NWR-0248-19-s04.pdf]

DV	Model η^2	IV	β (× 10^{-4})	SE (× 10^{-4})	t	p
Frontal MI (θ-low γ)	0.220	(Intercept)	–0.196	0.461	–0.425	0.670
		θ Amptd***	0.066	0.004	14.529	0.000
		Att state	–0.101	0.597	–1.688	0.091
		Interaction*	0.013	0.006	2.294	0.022
Visual MI (θ-low γ)	0.100	(Intercept)***	2.699	0.233	11.577	0.000
		θ Amptd***	0.024	0.002	9.902	0.000
		Att state**	–0.849	0.297	–2.864	0.004
		Interaction**	0.010	0.003	3.217	0.001
Frontal MI (θ-high γ)	0.008	(Intercept)***	8.657	0.641	13.496	0.000
		θ Amptd	0.011	0.006	1.747	0.081
		Att state	0.029	0.831	0.035	0.972
		Interaction	–0.011	0.008	–1.301	0.193
Visual MI (θ-high γ)	0.040	(Intercept)***	6.412	0.338	18.954	0.000
		θ Amptd**	0.009	0.004	2.648	0.008
		Att state	–0.168	0.613	–0.390	0.696
		Interaction	–0.001	0.007	–0.121	0.904

Note that all of the models were statistically significant, Fs > 6.030, ps < 0.001. Multicollinearity measured by variation inflation factors varied from 1.002 to 1.004 without interaction term, and from 2.432 to 13.037 with interaction term. θ Amptd: θ amplitude, Att state: attentional state; *$p < 0.05$, **$p < 0.01$, *** $p < 0.001$.
